# Witnessing the elimination of magic wands

**DOI:** 10.1007/s10009-015-0372-3

**Published:** 2015-03-31

**Authors:** Stefan Blom, Marieke Huisman

**Affiliations:** University of Twente, Enschede, The Netherlands

**Keywords:** Formal methods, Program verification, Correctness proofs, Mechanical verification, Specification techniques, Separation logic

## Abstract

This paper discusses static verification of programs that have been specified using separation logic with magic wands. Magic wands are used to specify *incomplete resources* in separation logic, i.e., if missing resources are provided, a magic wand allows one to exchange these for the completed resources. One of the applications of the magic wand operator is to describe loop invariants for algorithms that traverse a data structure, such as the imperative version of the *tree delete problem* (Challenge 3 from the VerifyThis@FM2012 Program Verification Competition), which is the motivating example for our work. Most separation logic-based static verification tools do not provide support for magic wands, possibly because validity of formulas containing the magic wand is, by itself, undecidable. To avoid this problem, in our approach the program annotator has to provide a *witness* for the magic wand, thus circumventing undecidability due to the use of magic wands. A witness is an object that encodes both instructions for the permission exchange that is specified by the magic wand and the extra resources needed during that exchange. We show how this witness information is used to encode a specification with magic wands as a specification without magic wands. Concretely, this approach is used in the VerCors tool set: annotated Java programs are encoded as Chalice programs. Chalice then further translates the program to BoogiePL, where appropriate proof obligations are generated. Besides our encoding of magic wands, we also discuss the encoding of other aspects of annotated Java programs into Chalice, and in particular, the encoding of abstract predicates with permission parameters. We illustrate our approach on the tree delete algorithm, and on the verification of an iterator of a linked list.

## Introduction

Verification of sequential programs with pointers has significantly profited from the advance of separation logic. Separation logic is an extension of classical Hoare logic that allows compositional reasoning about the heap, by explicitly considering only the part of global memory that is relevant for a specification [1]. In particular, heaps are modeled as partial maps from locations to values. Heaps can be composed when their domains are disjoint. This makes the logic highly suitable to reason about pointer structures, because it allows one to reason concisely about heap locations and (absence of) aliases.

The main contribution of this paper is that it discusses how two advanced features of separation logic, namely *abstract predicates with parameters*, and the *magic wand operator*, can be encoded in basic separation logic, thus making it possible to use existing separation logic verification tools that do not support these advanced features. We also discuss how these transformations are implemented in our VerCors tool set [2] and provide a few examples of verified code.

### Basic separation logic 

In classical Hoare logic [3], a program is annotated with properties about its intermediate states, where states consist of a stack and global variables. States are modeled as a *global store*, which is a mapping from variable names to values. In separation logic, the state consists of a stack and a heap. The stack is modeled as a store and the heap as a partial map from locations to values.

The main challenge when reasoning about heaps is *aliasing*, i.e., the possibility that two variables point to the same location on the heap. To handle this challenge, separation logic restricts access to the heap only to locations for which this access is explicitly allowed, i.e., the logic ensures that a program can only access a location for which it has an access permission. The key property of separation logic is that heaps can only be composed when they are disjoint. In particular, the *separating conjunction* of two formulas over the heap is only true if the heap can be divided into two parts, such that the first formula holds for the first part and the second formula holds for the second part. Therefore, if at some point in the program, it has access to both the location pointed to by variable $x$ and to the location pointed to by variable $y$, respectively, then $x$ and $y$ cannot be aliases, i.e., they cannot refer to the same location. The standard mathematical notation for this separating conjunction operator is $\mathrel {\star }$, and our textual notation is **.

Explicit access to a heap location is not only needed to evaluate a variable in executable code, but also to evaluate variables in specifications. A specification formula is only well defined, if there are appropriate access permissions to evaluate it, i.e., the formula should be *self-framed*. Most separation logics employ a syntactic restriction that immediately guarantees that every formula is self-framed: field names cannot occur anywhere in formulas, except as the first argument to a points-to predicate $x \mapsto v$, meaning that one has access to the heap location denoted by $x$, and that this location contains the value $v$. However, in this paper, we use the syntax of Implicit Dynamic Frames [4], which was incorporated into a separation logic called Total Heaps Permission Logic (TPL) [5]. In these two logics self-framing is not guaranteed by the syntax. Instead, formulas contain explicit access permission expressions, and are only well defined when sufficient access permissions are provided.

In addition, access permissions are defined as fractional permissions. This allows one to explicitly distinguish between read and write access to a location. This distinction is in particular useful to reason about concurrent programs, but can also be meaningful for sequential programs, because it provides support to specify that a data structure is immutable. The examples in this paper are all sequential, but since they are annotated in the language of the VerCors tool set [2], which ultimately targets the verification of concurrent programs, they are annotated using fractional permissions.

### Advanced separation logic

Besides access permissions and the separating conjunction, several more-advanced separation logic constructs have been developed, such as abstract predicates and magic wands.

*Abstract predicates* Commonly used extensions of separation logic are *abstract predicates* [6]. An important purpose of abstract predicates is to add *recursive* definitions to separation logic formulas, making it possible to define and reason about access permissions on unbounded data structures, such as linked data structures. Abstract predicates also provide control over the visibility of specifications, which allows one to encapsulate implementation details. Another useful feature of abstract predicates is that they can be declared without providing a definition (similar to abstract methods, e.g., Java). This allows one to use abstract predicates as tokens in specifications. This is used for example to specify behavior of a program as an abstract state machine, e.g., to specify mutual exclusion by requiring a token which is unique and thus cannot be held by more than one thread.

Since abstract predicates can be used to represent tokens, they define more than just a set of access permissions. Therefore, we will use the general term *resource* when referring to abstract predicates and/or access permissions.

Abstract predicates can have parameters, which can be program variables or fractions. The latter can be used, for example, to specify different access permissions to different parts of a data structure.

*Magic wands* Another feature of separation logic is the *magic wand* operator, also known as the *separating implication*, usually written  (or -* in our textual notation). Intuitively, the formula  is a resource that allows the *required* resource $\phi _1$ to be replaced by the *ensured* resource $\phi _2$ *once*. Thus, given a heap for which both $\phi _1$ and  holds, these formulas can be *consumed*, such that a formula $\phi _2$ is produced. This replacement is sometimes called *applying* the magic wand. This is in contrast to the normal implication $\implies$ (or **==> **), for which $\phi _1 \implies \phi _2$ is a boolean claim that states that $\phi _1$ can be replaced by $\phi _2$ an unbounded number of times. In particular, if for a given heap $\phi _1$ and $\phi _1 \implies \phi _2$ holds, then we can conclude that for this heap $\phi _1$, $\phi _2$, and $\phi _1 \implies \phi _2$ holds.

One of the applications of the magic wand is to specify loop invariants for iterative algorithms that explore data structures: knowledge about the current location in the data structure can potentially be exchanged for knowledge about the complete data structure explored so far, which is useful for programs that start at the entry point to a data structure, iteratively search for the correct place and then read and/or modify the structure. Before returning, suitable permissions and properties on the entry point must be re-established. The magic wand can naturally express that such a change is possible and applying the magic wand will perform the change. We show how this is done, by giving a loop invariant with a magic wand for challenge 3 from the VerifyThis@FM2012 Program Verification Competition [7]. This challenge is to verify an iterative tree delete algorithm: the removal of the element with the minimal key in a binary search tree.

In the literature, several other examples that illustrate the usefulness of the magic wand operator can be found, e.g., to specify an iterator protocol [8, 9] (discussed in Sect. 6.2), to reason about sharing in data structures [10], and to specify several common object-oriented design patterns [11]. The magic wand also shows up in other places, such as type systems [12].

### Separation logic tool support

There are several tools that allow reasoning about programs annotated using separation logic, or its variants, such as Implicit Dynamic Frames [4]. Among the most prominent are VeriFast [13, 16], SmallFoot [14], jStar [15], Chalice [17], Carbon [18], Silicon [19], and our own VerCors tool set [2]. All of these tools support basic separation logic, many support abstract predicates, but magic wands typically have limited or no support. We first briefly discuss the support provided by the different tools, and then the next subsection discusses how we add support for these advanced features in our own VerCors tool set.

SmallFoot and jStar support basic separation logic, without fractional permissions, as described above. They both support predicates, but neither supports the magic wand operator.

VeriFast supports reasoning about concurrent Java and C programs, and combines separation logic with fractional permissions. VeriFast provides support for abstract predicates with arguments. However, it provides no support for full magic wands; it only provides support for *lemmas*, which have the same functionality as the normal implication in separation logic (see Sect. 7.1 for more information).

Chalice supports reasoning about a limited object-oriented, concurrent language, using a special separation logic-like specification language, featuring explicit access permissions. It generates proof obligations to ensure that the annotations are self-framed. Further, it provides support for abstract predicates without arguments, except for the implicit **this** argument. It provides no support for magic wands.

Carbon and Silicon are the verifiers of the Viper project [20, 21]. Carbon is similar to Chalice and employs verification condition generation through Boogie. Silicon is similar to VeriFast and employs symbolic execution. An experimental version of the Silicon tool exists, which supports magic wands [22]. The basic approach to magic wands taken by Silicon is the same as the one employed in this paper. The difference is in how the basic approach is applied. The Silicon developers are aiming at a solution which is highly automated, whereas our solution aims at generality both in term of ability to specify programs and the ability to use multiple back-end verification tools. A more technical comparison can be found in Sect. 7.1.

In general, the validity of a magic wand formula is undecidable [23]. This leaves several obvious paths to work towards a solution: look for a suitable subset which is decidable, look for heuristics that are good enough in practice or, as we have chosen to do, require the program annotator to provide extra information about the correctness of the use of the magic wand formula.

### Contribution

*The VerCors tool set* As mentioned above, we extended the VerCors tool set with support to reason about abstract predicates with parameters and magic wands. VerCors supports the verification of concurrent (Java) programs, using permission-based separation logic, i.e., separation logic extended with fractional permissions to distinguish between read and write access.

For the design of our tool set, we leverage existing verification technology. Specifically, we implemented VerCors as a tool that compiles its input into the input language of a back end, then uses the back end to do the actual verification, and finally translates any error messages or warnings back to the input program. We use Chalice as a back end, because it allows for a relatively straightforward encoding of most separation logic constructs.^1^

Our specification language has been designed to remain as close as possible to the JML specification language for sequential programs [24], but with several separation logic extensions to make it suitable to reason about multi-threaded pointer programs. As mentioned above, in particular, the VerCors tool set supports abstract predicates with parameters and magic wands. However, the Chalice back end does not support this, and therefore this paper presents the transformations that we developed to support reasoning about these advanced separation logic constructs in VerCors.

- The first transformation converts a program with predicates with arguments to a logically equivalent program with predicates without arguments.
- The second transformation takes a program with magic wands and converts it to an equivalent program without magic wands.

These two transformations are the main contribution of this paper.

*Approach* To define the transformations, we introduce the notion of a *witness* objects that encode instances of abstract predicates with arguments and magic wand formulas.

The witness object for an abstract predicate instance stores the values of the predicate arguments. The witness class defines a predicate without arguments that holds for a witness object if and only if the original predicate instance with arguments holds.

The witness object for a magic wand encodes a description of how to convert the required resources of the magic wand into the ensured resources. To apply this conversion, a magic wand witness defines a predicate that specifies the additional resources that are needed to perform this conversion once. This predicate is encoded into the witness object for magic wands in the same way as it is done with witness objects for predicates. When a magic wand witness is generated, the user has to provide a *proof script* to define: (i) a *constructor* that behaves in the same way as a magic wand introduction rule in separation logic, and (ii) an *apply* method that behaves in the same way as a magic wand elimination rule in separation logic. To specify this proof script, VerCors provides a dedicated proof annotation syntax. Notice that, because the user is required to provide a proof script, it no longer matters whether the *existence of a proof* is decidable or not.

We also use the proof script to construct our encoding in such a way that the result of executing the original program is the same as executing the encoded program. Due to this *executability*, our transformation can not only be used for static checking, but also for run-time checking. Moreover, it has the potential to be extended into a translation from programs specified in separation logic into programs specified in first-order logic. This would allow alternative static checkers to be used, and it would also provide a way of using first-order logic run-time checkers for separation logic (by transforming a program annotation with separation logic into a program specified with first-order logic, and then using a run-time checker for first-order logic).

It should be noted that the first transformation is similar to a transformation that has already been described by Jost and Summers [25], in a paper that considers translating VeriFast specifications into Chalice. Our transformation is slightly more complicated, but also slightly more general. A more technical comparison can be found in Sect. 7.1.

Finally, we would like to emphasize that our solution to the verification problem of the magic wand was inspired by the Curry–Howard isomorphism [26], which turns a verification problem into a type-checking problem by encoding a proof as a program. This intuition is further emphasized by the way that we write annotations: formulas are typically manipulated using logical rules, while witnesses are manipulated by methods defined on them. Thus, the encoding of magic wand formulas in this paper transforms the program verification problem into the programmatic manipulation of specification-only (or ghost) objects. We believe that this approach is attractive for software engineers who are already comfortable with an imperative way of thinking about program behavior, but may need to invest a lot of time to get comfortable with logical manipulation of complex formulas.

*Overview* The remainder of this paper is organized as follows. First, we provide a more detailed introduction to our variant of separation logic, and how this is supported in Chalice and the VerCors tool set. Section 3 presents the tree delete challenge of the VerifyThis 2012 competition and discusses an intuitive solution for the challenge that uses a magic wand. Section 4 focuses on the encoding of predicates with parameters. We continue in Sect. 5 with the elimination of the magic wand. Then in Sect. 6, we present machine-checked versions of the challenge and of an additional example, namely an iterator protocol. Finally, we conclude and discuss related and future work.

## Background

This section introduces the logical formulas that we use in this paper, and how this is concretely supported by the VerCors specification language. We also discuss a few extra annotations that help the proving process. Then, we briefly introduce the Chalice tool, which is the target of our transformation, and the overall architecture of our VerCors tool set.

### Deterministic separation logic

Our logical formulas are based on those in Total Heaps Permission Logic (TPL) [5], which is a merge of separation logic and Implicit Dynamic Frames [4]. Compared to TPL, we have added infinitesimal permissions and predicates [6], while we restrict to disjunction-free resource formulas. We will call our logic *Deterministic Separation Logic* (*DSL*).^2^ As semantics for this logic, we use Implicit Dynamic Frames with fractional permissions. That is, our semantic framework is that of Chalice [28].

Our notion of infinitesimal permission corresponds to what is called an infinite supply of infinitesimal permissions in the underlying theory of Chalice [28]. That is, the infinitesimal fraction $\epsilon$ is a permission value that is non-zero, so it suffices for reading, but it is smaller than any fraction. In practice, this means that any location for which we hold an infinitesimal permission is immutable.

To keep the resources specified with the logic deterministic, we eliminated all sources for non-determinism, such as disjunction, existential quantification and negation over resource formulas. This restriction only applies to specifications of resources. We do allow negation, disjunction and existential quantification over values. To achieve this, we define two classes of formulas: boolean formulas ($B$, typical elements $b_i$) and resource formulas ($R$, typical element $r_i$). Boolean formulas state properties over values only and can be written in full first-order logic. Access permissions can only be used as part of resources formulas, which includes all boolean formulas, and can be combined using separating conjunction and magic wand. Note that, the separating conjunction on resources is not commutative, as Chalice requires all formulas to be self-framed.

*DSL as VerCors specification language* The VerCors program specification language is DSL, but with some convenience syntax added. In addition, we introduce a few useful VerCors annotation constructs.

VerCors specifications are embedded in comments using syntax that is borrowed from JML [24]. That is, all comments that start with a **@** are part of the specification. This holds for both single-line (**//@ ...**) and multiple-line (/*@ ... */) comments. Table 1 gives the complete grammar of our property annotation language.

**Table 1:**
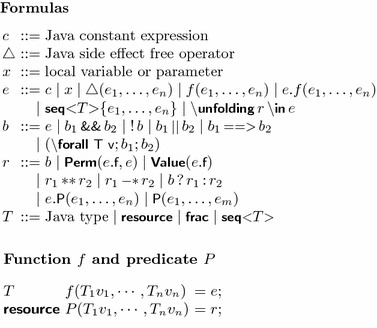
Grammar of the VerCors specification language

Note that, if a method contract contains multiple **requires** clauses then that is equivalent to requiring the separating conjunction of those clauses. The same is true for **ensures**.

The VerCors tool supports the implication operator **==>** both as a boolean operator and with a boolean formula as the condition and a resource formula as the conclusion. The latter case is syntactic sugar, defined in terms of the if-then-else operator: 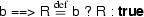 In addition, VerCors provides syntactic sugar for the following common specification pattern for predicate : 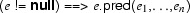 This construct will be abbreviated as: 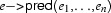


In addition to Java’s standard primitive types, the specification language has two additional primitive types: **resource** and **frac** to type permission and fraction expressions, respectively. As in Chalice, the domain of **frac** is a value between $1$ and $100$, where $100$ means a full write permission and any value less than 100 denotes a read-only permission. This restriction is made because we use Chalice as a back end, not because the encoding requires it. In principle, the techniques described in this paper work over any separation algebra [29]. Note that, the syntactic domain of **frac** does not include the infinitesimal fraction $\epsilon$. Hence, we denote permission $\epsilon$ on the location $l$ by .

For specification convenience, the tool also provides a built-in polymorphic list or sequence type , where  can be any type (not necessarily a class). This type translates directly to the Chalice type of the same name. The syntax for a constant list borrows from the syntax for a constant array, e.g., the list  is written as 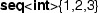 Several standard operations on sequences are available. Given sequences , we have:

- concatenation: ;
- first element: ;
- other elements: ; and
- length: .

Only expressions that do not have observable side effects are safe to use in specifications. Such expressions are called *pure*. In JML, it is possible to specify and verify that a method is pure. The VerCors tool supports this too, but within the context of this paper, we will limit ourselves to *functions*. A function is a method that simply returns an expression. Functions are defined inductively, ensuring that their evaluation always terminates.

**Fig. 1:**
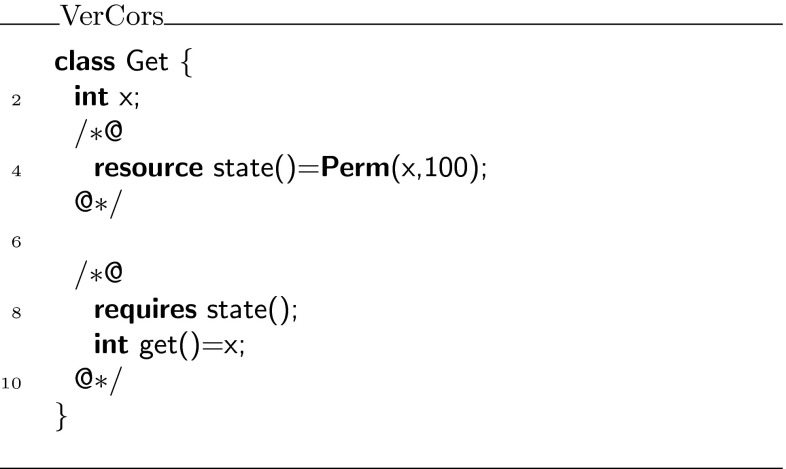
Simple getter example

Because functions can only return expressions, we use an abbreviated syntax for them. The formal parameter declaration uses the same syntax as a Java method declaration, but instead of the method body we write **=** followed by the expression that defines the function. The same syntax is used for predicates. For example, in Fig. 1, line 4, we first define the resource predicate , which defines write access to field . Then, we define the function  that returns  in lines 8, 9. Note that, the function  requires the  predicate to be allowed to access . Also note that although it is not explicitly written, the function  does in fact ensure  too, because by definition functions cannot lose resources.

In method contracts, we will often employ ghost parameters and ghost return values. These are declared by **given** and **yields** clauses that precede the method declaration. For example, an integer ghost parameter  and a boolean ghost return value  for a method **m** are specified as: 
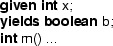
 Implicitly, a method contract is universally quantified over the variables in the **given** clause, and existentially quantified over the variables in the **yields** clause, similar to how a normal method contract is universally quantified over its parameters and existentially quantified over its return value.

When a method with ghost parameters is called, the parameters must be passed and the results may be stored. For this purpose, we use the keywords **with** followed by a block, and keyword **then** followed by a block. A parameter is passed by setting it in the **with** block. A result is received by assigning it to a ghost variable in the **then** block. For example, invoking method  with $37$ as the ghost argument and storing the ghost return value in a ghost variable  is written as 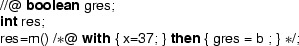


When using predicates, there is no semantic difference between a predicate invocation and an instantiation of its definition. However, automatic provers cannot simply replace defined objects by their definitions, as this would lead to an infinite search space, which might cause the prover to become non-terminating. As a consequence, in many cases the equivalence between a defined object and its definition cannot be proven automatically. To overcome this limitation, VerCors supports proof hints that tell the prover to explicitly convert between the two forms. The operation of replacing a predicate invocation by its definition is called **unfold**, and the reverse operation is called **fold**. Sometimes, it is necessary to unfold a predicate temporarily in an expression. The syntax to do that is .

This completes the overview of the features of the specification and annotation language that we need to discuss the witness transformations in this paper. The tool supports more features. For example, in boolean formulas both existential and universal quantifications have been added with the same syntax as JML, and in resource formulas, universal separating conjunction has been added to be able to make statements about permissions to elements of arrays. The tool also supports inheritance using the theory of abstract predicates and inheritance by Parkinson and Bierman [30], but cannot use the full power of that theory because specifications are restricted to monotone predicate families, as introduced by Haack and Hurlin [31, 32].

In the examples below, whenever necessary, we will explain more details of the specification syntax.

### Chalice

As mentioned above, Chalice is a tool for the verification of concurrent programs [17]. Its input language is a simple object-oriented language with built-in specification features. Features of the Chalice language are basic classes (no static members or inheritance), fields and three kinds of ‘methods’:

- standard methods, which can be used in executable code;
- functions, i.e., to evaluate a property about the state, which cannot have side effects, and can, therefore, be used both in executable code and in specifications; and
- predicates, which specify the access permissions that both methods and functions require and can only be used in specifications.

The specification language of Chalice supports field permissions in the same way as DSL above, albeit with a different syntax (**acc** instead of **Perm**,  instead of **Value**, and  is used as a connective for permissions, instead of ). Standard boolean expressions and functions can be used in specifications. In addition, Chalice has support for (recursive) predicates. However, these predicates cannot have explicit parameters, i.e., they are limited to the implicit parameter **this**. Both functions and predicate definitions should terminate.

In Chalice, all unfoldings of predicates in Chalice functions must be explicitly written. For example, the  function in Fig. 1 is translated to the Chalice code 
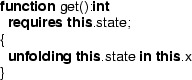
 Folding and unfolding of predicates in method bodies in Chalice are done using the same keywords as for VerCors.

The Chalice tool verifies annotated code by generating an annotated Boogie program [33], for which the Boogie verifier subsequently generates first-order logic proof obligations.

### Architecture of the VerCors tool set

As mentioned above, the VerCors tool set leverages existing verification technology, and encodes annotated concurrent Java programs into Chalice and Boogie.

Input for the tool is source code annotated with method contracts. These contracts are translated via Chalice and Boogie into the formalism used by a back-end prover. The diagnostic messages provided by the back-end tool are then (partially) reverse engineered to provide diagnostic output messages to the user. Each failure can optionally be accompanied by the full details provided by the underlying verification engine.

**Fig. 2:**
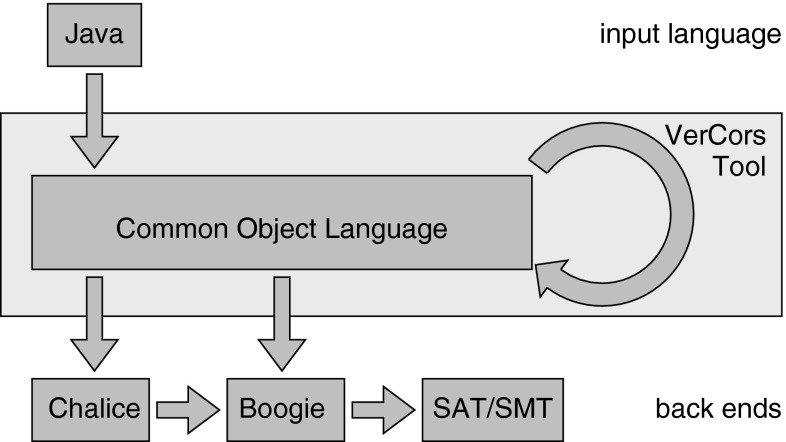
VerCors tool set architecture

The VerCors tool set is built along the classical pattern of a compiler. That is, the input programs are parsed into an abstract syntax tree on which several transformations are applied before they are passed on to one of the back ends. The arrows in Fig. 2 indicate the possible paths a problem can take from input to solver. They reflect that Chalice works by translating its input into Boogie and Boogie in turn works by generating a problem for an SMT solver, such as Z3 [34]. The direct arrows from the intermediate format (Common Object Language) to Chalice and Boogie indicate that (depending on the precise verification task) the tool will transform programs into input programs for Chalice, or for Boogie directly. In this paper, we only consider the encoding via Chalice.

## The tree delete challenge

As a motivating example for our work, we use the iterative removal of the element with the least key from a binary search tree, i.e., challenge 3 from the VerifyThis@FM2012 Program Verification Competition [7]:

Given: a pointer t to the root of a non-empty binary search tree (not necessarily balanced). Verify that the procedure in Fig. 4 removes the node with the minimal key from the tree. After removal, the data structure should again be a binary search tree.

**Fig. 3:**
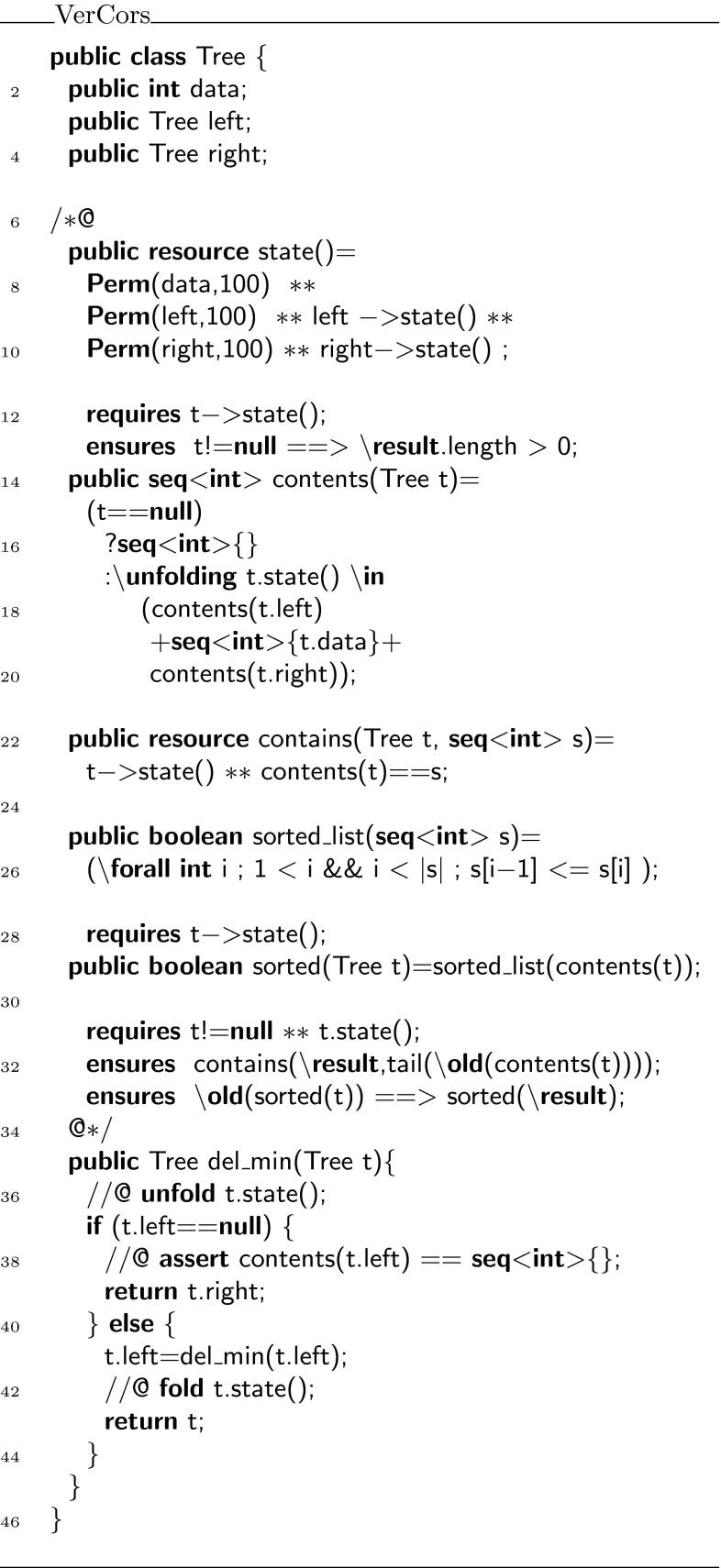
The recursive implementation of minimal element deletion

In Fig. 3, we show the fully specified listing of the recursive solution of the problem. We will discuss that listing and then sketch the iterative solution.

Input for the tree delete algorithm is a binary search tree, in our case this tree is represented in the class  with an integer data field and left and right sub trees (lines 2–4). The goal of the algorithm is to delete the element with the smallest key, i.e., the left-most node from the tree, and the challenge is to prove that the resulting tree remains a binary search tree.

To provide a specification for the algorithm, we first add the following definitions to the  class:

- the predicate , representing permissions to the field locations making up the tree (lines 7–10);
- the function , capturing the list of integers stored in the tree (lines 12–20);
- the predicate , expressing the permissions and the stored values simultaneously (lines 22–23); and
- the functions  and  stating that the represented list is sorted (lines 25–29).

The  predicate defines the permissions on the tree. If one holds the  predicate, one has write permission on the fields ,  and , and recursively also on the subtrees pointed to by  and , provided they are not **null**. The pure method  defines the contents of a tree to be the contents of the nodes’ data fields, read from left to right.

Using these specifications, the method , which implements the deletion of the element with the minimal key is specified in lines 31–33. This contract states that given a non-empty tree, the algorithm removes the first element of the list that is represented by a tree. Moreover, if the input represents a sorted list then the output represents a sorted list too. From this, we can derive that the requirements of the challenge hold using a few well-known facts about binary search trees. First, for a binary search tree the list it represents is ordered. Therefore, removing the first element is the same as removing the least element. Moreover, the permissions used are full write permissions, which implies that the underlying linked data structure is tree shaped. If  holds on a node, we have full permission on the data field of that node. If the same node occurs twice in the tree (or on a cycle), then we would have two full permissions (or more) on that node. That is impossible, so there cannot be any shared node in the data structure, see also [35].

**Fig. 4:**
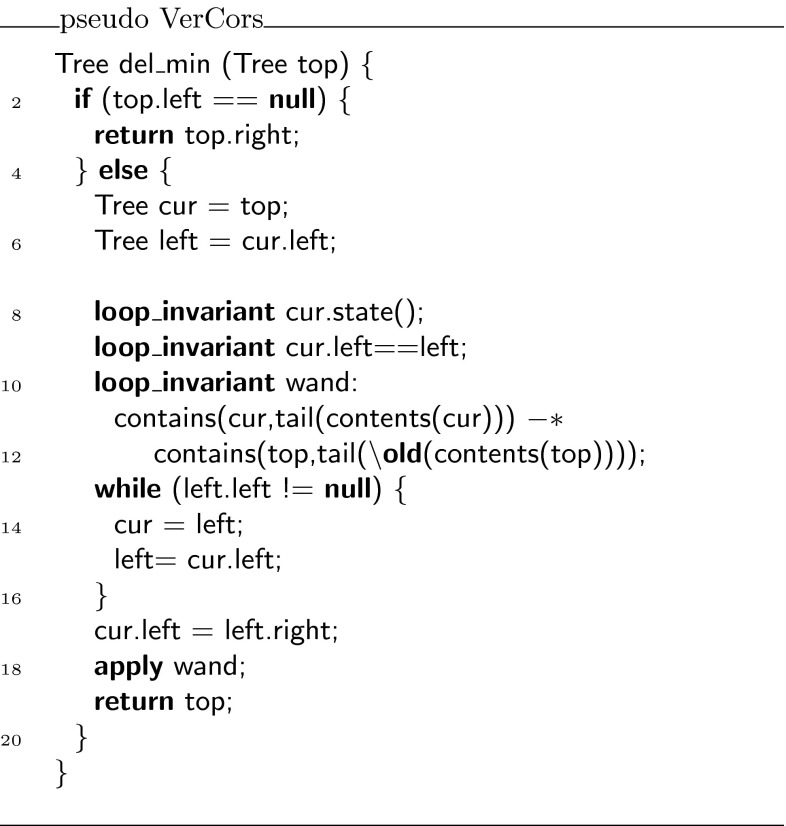
The iterative implementation of minimal element deletion

Finally, lines 35–45 of Fig. 3 contain a recursive implementation of this algorithm. It is easy to see, and to verify, that this implementation respects the specification of . To illustrate this, we have decorated this recursive implementation with the annotations that are needed for the VerCors tool to verify that this implementation respects the specification above: essentially all that is needed is opening of the  predicate at the beginning of the method, closing of the  predicate at the end of the method body, and an explicit assertion that if  is **null** then the contents of  are the empty list. The assertion, while logically superfluous, guides the underlying prover towards the solution. Without it, verification results in an inconclusive verdict due to a timeout.

In contrast to the recursive version, the verification of the iterative version of this algorithm, as requested by the actual challenge description, is much more involved. One needs to specify an appropriate loop invariant that retains all permissions on the entire tree data structure during the iterations that compute the left-most node in the tree. The invariant must be written in such a way that the deletion of the left-most node afterwards is allowed and that the permissions on the whole tree can be recovered.

The core of the problem is the treatment of permissions, which are given in the form of a tree. In each iteration, the focus on the tree (i.e., the variable ) is shifted by one step. However, once you have reached the left-most node, you want to move back the focus to the root of the tree, i.e., after the loop has finished, the method should continue with access to the root of the tree. The magic wand is highly suited to handle this: all the permissions on the traversed path (including all its unvisited subtrees) are stored “inside” the magic wand, and by giving up the focus on the current node, focus on the root can be retrieved. That is, we build a magic wand that allows exchanging full permission on the tree with the focus on the current node by full permission on the tree with the focus on the root.

Figure 4 contains the iterative implementation of the tree delete algorithm, with the key loop invariants necessary to verify this method. Note that, rather than using the competition version verbatim, we have eliminated a superfluous variable and renamed all variables to make the code more understandable. Also note that while our ultimate goal is to further develop the tool set in such a way that this code can be verified essentially as it is written in Fig. 4, the current version of our tool set needs a lot more annotations. The fully annotated version, which can actually be verified with the VerCors tool set, can be found in Fig. 18 (on page 20).

The variables in the algorithm denote the following:

is the pointer to the root of the complete tree;
is the currently explored node; and
is the left subtree of .

The loop invariant expresses the following:

- the program holds permissions for the currently explored node ( predicate);
- the currently explored node is the root of a tree ( predicate);
is the left subtree of ; and
- a promise (by means of a magic wand) that if  is modified to represent a tree with the left-most element removed, and if the program holds access permissions to this tree, then this can be exchanged to access permissions on a larger tree, which also has the left-most element removed compared to the tree at the start of the procedure.

With this loop invariant, we can correctly capture sufficient “promises” about the rest of the data structure to verify correctness of the algorithm.

To make this proof amenable to automated tool support, we need an encoding of the magic wand formula using classes, separating conjunction and the points-to predicate. To be able to do this encoding, the user has to provide some evidence that the magic wand is indeed correct, i.e., that the required resources can indeed be exchanged for the promised resources. We introduce this encoding in two steps. First, we introduce the basic idea behind encoding formulas as objects using the witness encoding of predicates with parameters. Second, we show how magic wands can be encoded in a similar style. Finally, we show how we can use this approach to complete the verification of the tree delete challenge.

## The encoding of predicates

In this section, we show how predicates with explicit parameters, different from the implicit **this** parameter, are encoded in Chalice. This problem was also considered by Jost and Summers as part of their translation from VeriFast to Chalice [25]. In that paper, it is explained how some resource predicates with arguments can be rewritten as combinations of resource predicates without arguments and boolean functions with arguments. This does not always work, so they also have a way of encoding predicate arguments as fields of the original object. Our encoding goes one step further and encodes the arguments as fields in separate objects, called *witness* objects. The original predicate with arguments can then be redefined as a predicate on the witness object that refers to fields instead of referring to arguments and it thus needs no arguments. The reason that it is beneficial to have this more complicated version is that, if the arguments are encoded inside the object, it may be complicated to encode situations where different predicate instances must be held on an object. Such situations occur naturally in many styles of parallel programs, e.g., in divide-and-conquer style programs. Using a different witness objects for every instance held, we can handle this situation in a very simple way.

We describe the witness approach first for simple predicates, and then for recursive predicates. Then, we describe the encoding algorithm. Finally, we consider the soundness and completeness of the transformation.

### Predicate witnesses

**Fig. 5:**
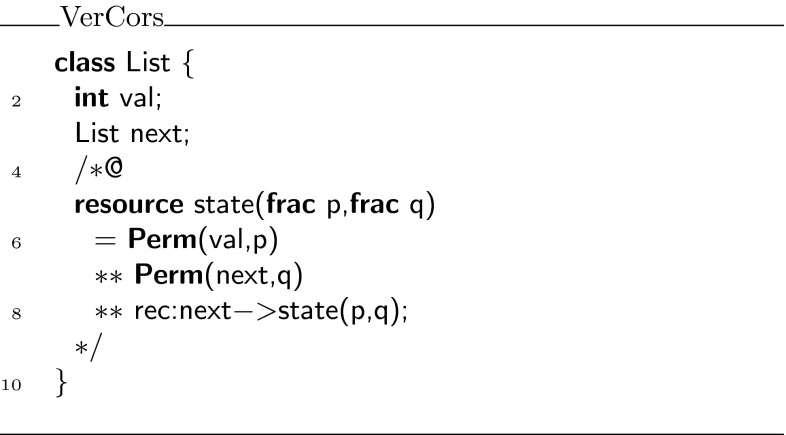
Recursive state predicate with permission parameters

Suppose, we wish to define a predicate that captures that you have fraction $p$ to access the elements stored in the list (via the  pointer), and fraction $q$ to traverse the  pointer to the next element in the list, as is done in Fig. 5. This predicate cannot be rewritten to fall within Chalice’s syntax for formulas. To get around this problem, we introduce the notion of *witness*. This witness is a carefully encoded object, containing a  predicate, which holds for the witness object if and only if the original predicate holds. Witnesses can be constructed and manipulated using Chalice (ghost) code.

#### Witnesses for non-recursive predicates

**Fig. 6:**
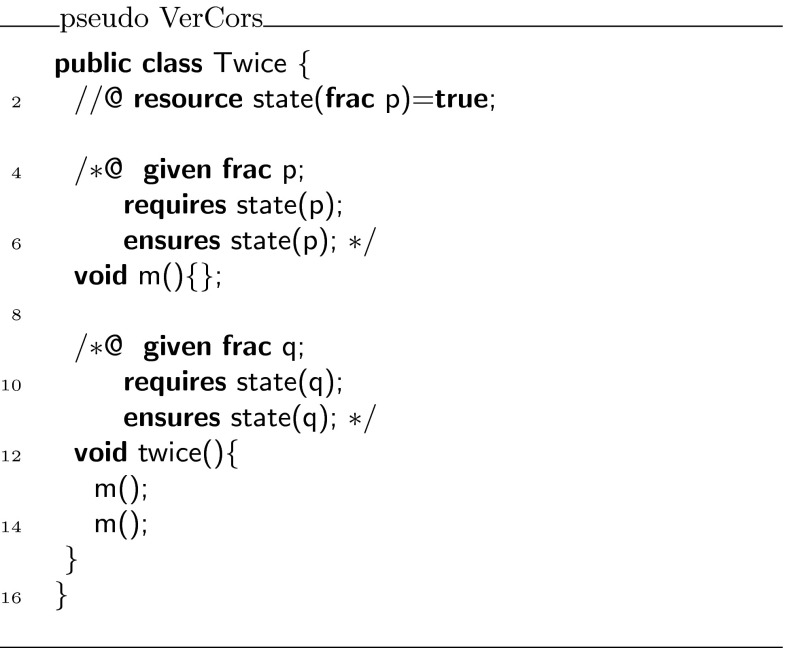
Simplified class

To describe how this witness object is constructed and reasoned about, we first consider the encoding of a very simple predicate, whose body is just true. Class  in Fig. 6 defines such a predicate, called .

**Fig. 7:**
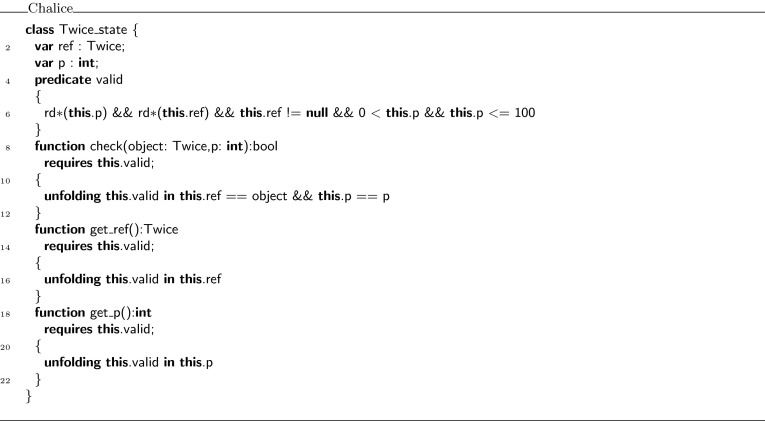
Chalice encoding of the witness class

Figure 7 shows the definition of the witness object in Chalice that encodes the  predicate declared in class . The witness object is an instance of the class . This class definition is generated by the VerCors tool set. The class has two fields:  refers to the object where the original predicate is defined, and  holds the value of the parameter . Furthermore, it defines a predicate  that encodes the original  predicate, but using the fields of the witness object, instead of the original predicate’s parameters. In addition, the class contains a function  that compares the predicate parameters in the original specification with the values in the fields of the witness object, and expresses that they are the same. If  is the witness object for a predicate , then an assertion  becomes essentially  ${}\mathrel {\star }{}$  in the encoding (see for example Lines 3, 4, 9, and 10 in Fig. 9). To complete the description of the class , it also defines getter functions for all fields in the class.

Using the witness object of the predicate, we wish to show that class  in Fig. 6 is correct. For a human, it is easy to see that the body of  satisfies its contract: the pre- and postconditions of  and  are all the same, and the calls to  thus can be put in sequence.

**Fig. 8:**
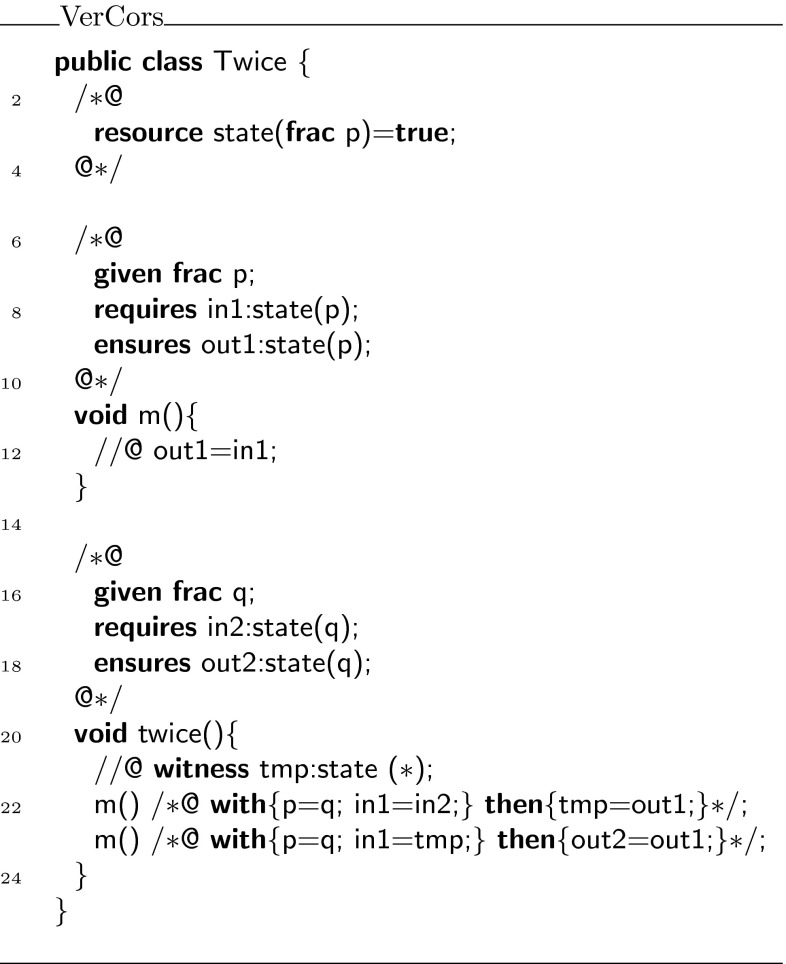
Fully annotated class

To ensure that the tool can establish the correctness of , we need to decorate it with some additional proof annotations, as shown in Fig. 8. First of all, every usage of predicate  has been prefixed with a label, which refers to a witness (an instance of the class  after encoding). Thus, for example in lines 8 and 9,  is used to refer to the witness object for predicate  that is passed as argument to the call of , while  is used to refer to the witness object for the predicate returned by this call.

To be able to refer within the body of method  to the witness object returned by the first call to , in line 21, we use the **witness** keyword and the same labeling notation to declare  as a variable that can refer to a witness. Note how the argument of the predicate is a wildcard , meaning that its value is irrelevant.

The **with** block instantiates the variables declared in the **given** block for the method (in this case ), and the witness object associated with the precondition of this call. The **then** block assigns the witnesses that are returned by the method call to the appropriate variables.

**Fig. 9:**
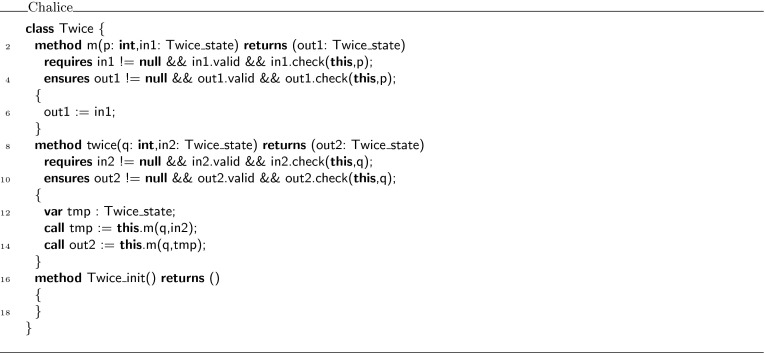
Chalice encoding of class

Finally, Fig. 9 shows the encoding of class  in Chalice. Notice how the  predicate is encoded as a combination of  and **check**. The information from the **with** block is used to generate parameters for the Chalice method calls. The information from the **then** block is used to generate assignments for the returned values of the Chalice method calls. Also notice that the witness names  and  have become an argument and a return parameter of the method , respectively (in VerCors syntax, these are ghost parameters declared with **given** and **yields**, respectively).

#### Witnesses for recursive predicates

**Fig. 10:**
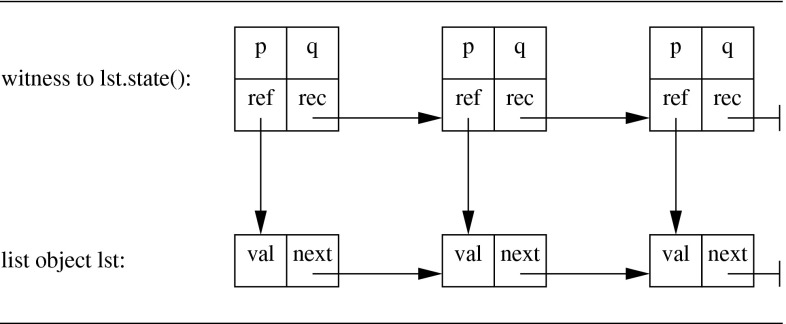
Example witness structure for linked list

**Fig. 11:**
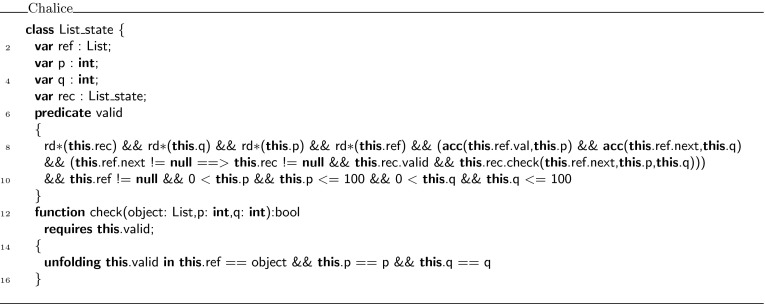
Fragment of the  witness encoding

When a predicate is recursively defined, such as the state predicate on linked lists in Fig. 5, the witness encoding results in an object that is recursive too. For every recursive invocation of the predicate in the predicate definition, the witness contains a field that refers to the witness that provides the evidence for the recursive call. Thus, the witness for a predicate being valid on an object is actually a tree of witness object instances, whose structure matches the calling structure of the evaluation of the original predicate. This tree is a finite object because in our restricted case predicate definitions always terminate. For example, in Fig. 10, the linked list of witnesses at the top reflects that the definition of  applied to the list of three elements at the bottom makes two recursive calls. The definition of the witness object is given in Fig. 11, where the field  refers to the witness object for the recursive call of the predicate. Note further how the conditional part of the  predicate in line 9 matches the conditional invocation  in the original predicate definition.

### Recipe for the encoding

As presented above, the encoding into Chalice generates a witness class for every predicate definition, replaces predicate invocations in logical statements by validity checks on witnesses, and adds getter functions for use by witness classes as well as variables that are needed to store witness objects.

The complete recipe for the encoding (as a VerCors to VerCors program transformation) is as follows:

1. Every predicate definition 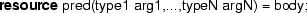 declared inside class  gives rise to the declaration of a sibling of , called , containing:
  a field  to refer to the object for which the validity of the original predicate is encoded;
  fields , ...,  to store the parameters of the original predicate;
  a predicate , whose definition is the separating conjunction of write access to the fields of the predicate class and the translation of the body of ;
  a function   that can validate if the reference and parameters match; and
  getter functions for all fields.
2. In the method specifications and other annotations, every predicate invocation   is replaced by the following separating conjunction: 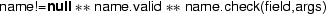 This encoding depends on name being defined, therefore, some additional declarations are necessary, depending on where the invocation occurred:
  in a requires clause: add a parameter  to the **given** clause;
  in an ensures clause: add a return value  to the **yields** clause; or
  in the body of a predicate definition: add a field  to the definition of  and also add  to the  predicate of the class .
3. The original class is modified as follows:
  the predicate definition is removed;
  every occurrence of 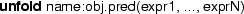 is replaced by ; and
  every occurrence of 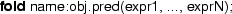 is replaced by the block 
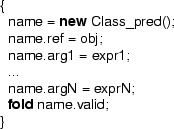

### Soundness and completeness of the encoding

The soundness of the witness encoding for predicates follows from the fact that ${\mathbf {\mathsf{{o.pred}}}}({\mathbf {\mathsf{{arg}}}}_1,\ldots , {\mathbf {\mathsf{{arg}}}}_N)$ is valid in a state if and only if , where $w$ is a witness of the former formula.

In our restricted setting (terminating predicates only), we also have completeness because witnesses can be constructed by induction on the call graph of the predicate invocation ${\mathbf {\mathsf{{o.pred}}}}({\mathbf {\mathsf{{arg}}}}_1,\ldots , {\mathbf {\mathsf{{arg}}}}_N)$ by following the recipe for the encoding described in the previous section. When using fixed-point semantics, not all predicates have finite call graphs. Hence, our method is not complete under fixed-point semantics for predicates.

## The encoding of magic wands

Now that we have seen how predicates with parameters are encoded in Chalice using witness objects, we discuss how magic wands are encoded based on a similar approach. The encoding requires the user to provide a proof script, which provides sufficient information to create a magic wand witness object and to apply it. The proof script language will also be discussed.

### General idea

The general idea behind our encoding is that a magic wand encapsulates two features. First, like a predicate, it can store resources. Second, like a lemma, it can describe how the stored resources combined with the additionally required resources (left-hand side formula) can produce the ensured resources (right-hand side formula). As in the previous section about predicates, the encoding results in a witness object.

Following that idea, what we want to do is as follows: each magic wand formula is encoded as an instance of the class . Assuming that we have a data type that can contain formulas, the formulas describing the required and ensured resources of the magic wand are fields of this class. A formula field is also used to describe the extra resource needed to transform the required resources into the ensured resources. In addition, a description of how the required resources can be exchanged for the ensured resources is stored in a field of type proof.

The extra resources are given to the constructor and *stored inside* the magic wand object (in the  field) by folding them into the  predicate, which is given back to the creator of the  object. The extra resources are now hidden and cannot be accessed directly any more. The only way to retrieve them is using the  method. The specification of the  method requires the required formula of the magic wand, as well as the  predicate and ensures the ensured formula, while its body is the exchange description.

**Fig. 12:**
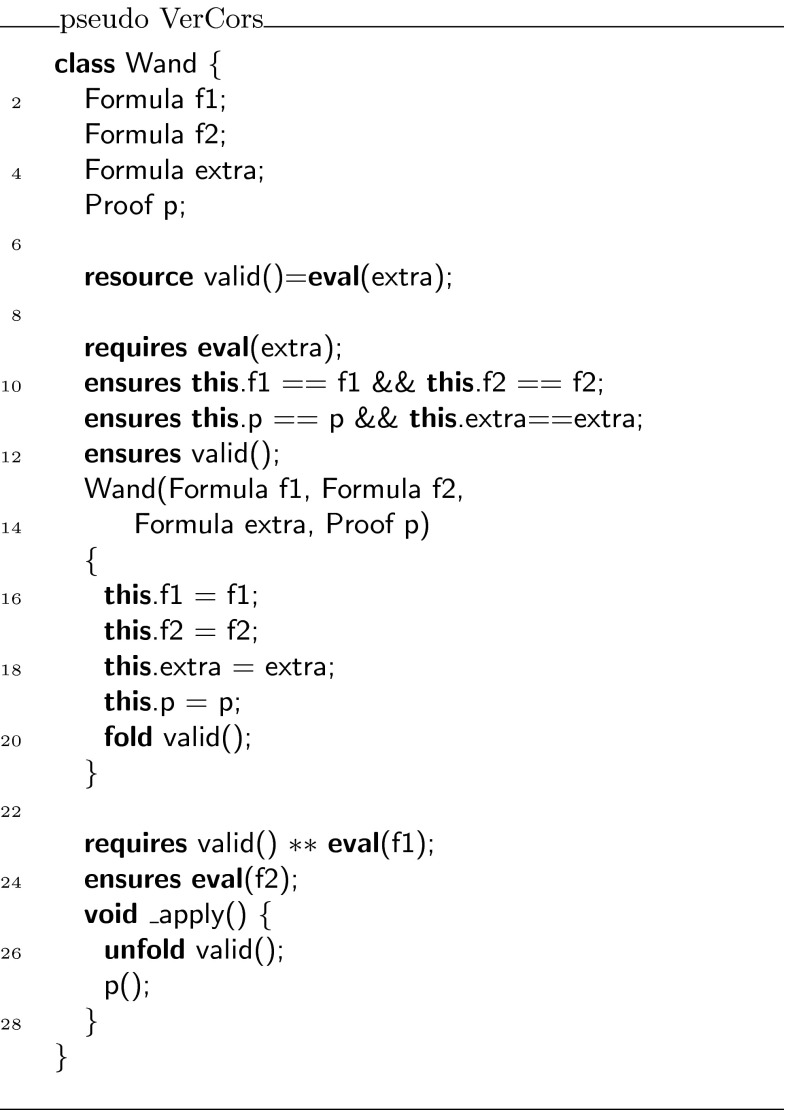
Idealized wand encoding

Correctness of the  method w.r.t. its specification ensures that the resources declared in the  field and stored in the  predicate of the wand, together with the required resources are sufficient to establish the ensured resources of the magic wand. Figure 12 shows the definition of this wand class. The precondition of the  method ensures that the method is invoked no more than once, because the  predicate has to be given up during the call.

Notice that this is not a valid encoding, as it uses types such as  and , which are not supported in the specification language. Neither is there support for the **eval** operator for evaluating a formula as part of a resource expression. Therefore, below we discuss how the ideas of this idealized encoding can be realized by a correct Chalice class.

### Encoding of magic wands in Chalice

This section discusses how the idea described above is used to generate a specific class for each type of wand formula that is used. Moreover, the proof script cannot be passed as a parameter, instead we encode it by an identifier, and generate a body of  that selects the appropriate proof script, depending on the value of the identifier.

**Fig. 13:**
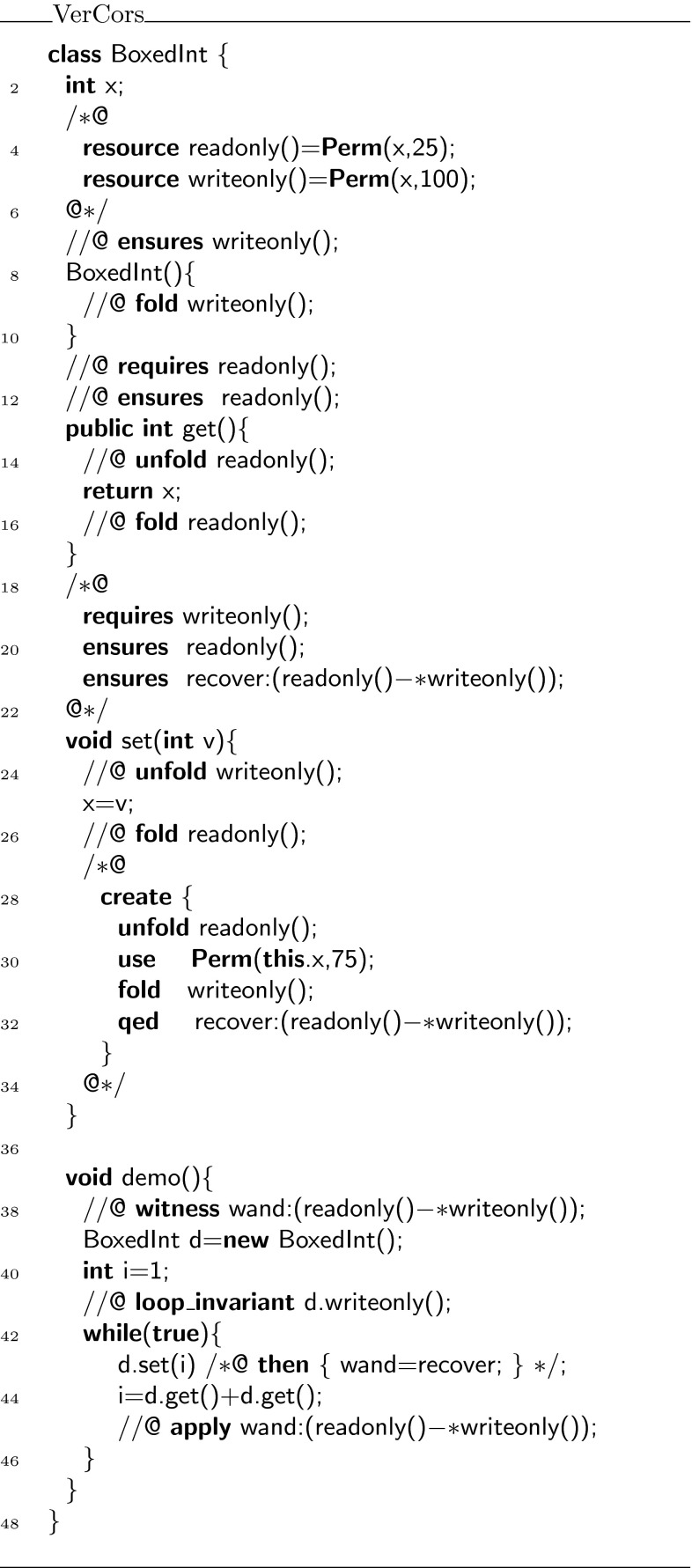
Class

Consider for example the  class in Fig. 13. This class implements a field , and setter and getter functions for that field. The contracts are written to enforce a non-standard set/get protocol,^3^ which was inspired by the iterator protocol of Hurlin and Haack [9]. Every  object can be in two states: read mode and write mode. When an instance is created, it is created in write mode. In write mode, the only method that can be called is . After setting, the object is in read mode. In read mode, the only method that can be called is , and the object stays in read mode. However, it is always possible to reset the object to a different value. To do so, write mode must be re-established. Hence, the  method ensures not just the predicate , but in addition also the magic wand , which can be applied to enable writing. Figure 13 also defines a method  to illustrate how the magic wand is used.

The code is mostly self-explanatory, but three elements are worth noting:

- Every magic wand is given a label, which is used to be able to identify which magic wand is addressed. In this example, the labels  and  are used (in lines 21 and 38, respectively).
- The syntax for creating a magic wand is 
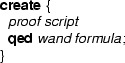
 To write the proof script, the usual proof hints (fold/unfold/etc.) are allowed, and in addition two new statements are introduced: **use** $R$  and **qed** $R$ . The **use** statement asserts that formula $R$ holds at the time the magic wand is created and stores the resources represented by this formula inside the magic wand. For example, the proof in lines 28–33 stores 75 % of the permission on the field  by means of the statement . The **qed** statement ends the proof script for the magic wand.

**Fig. 14:**
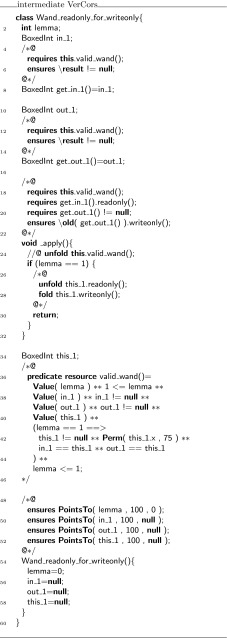
Encoding of the wand formula in class

**Fig. 15:**
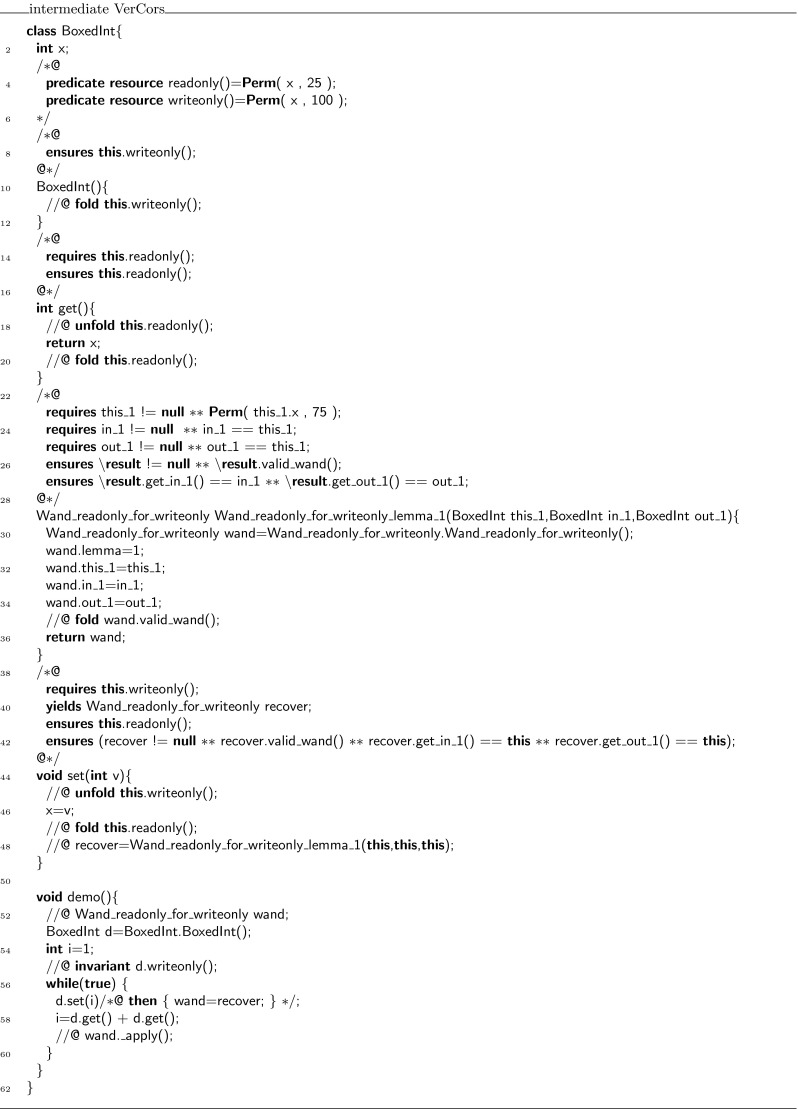
Class  after introducing witness objects

Our encoding declares a class for the witness object, called , as shown in Fig. 14, to represent the magic wand. Moreover, it rewrites the  class to replace magic wand formulas by manipulations of the witness object (see Fig. 15).

Instances of  are used as witnesses to the validity of all magic wands that match  for arbitrary expressions  and . Because in the encoding proofs are checked when a magic wand is applied and not when it is created, we number all proofs and store the proof number in the  field. Also, the values of  and  at the time of the creation of the magic wand are stored in the fields  and , which have matching getter functions. The values of other variables that are used in the proof are also stored in the object. In this case, the value of **this** for lemma 1 is the only value needed and it is stored in the field .

The contract of the  method (Fig. 14; lines 18–21 essentially requires the wand to be valid, and the  predicate on  to hold. It ensures the  predicate on . The actual code uses getters and has to deal with non-null issues as well.

The body of the  method consists of an unfold statement, followed by a case distinction over the proofs that have to be verified. For each proof, the proof hints that form the actual proof are copied into the corresponding branch in the  method. That is, the **use** keywords and the **qed** keyword are dropped. For example, from the body of the **create** in lines 28–33 in Fig. 13, only the unfold and the fold are copied to the body of the  method in lines 23–32 in Fig. 14.

The  predicate specifies (write) access to all fields in the object and for every proof it has a conditional requirement that all of the required permissions and properties hold. For our example, this means that for example if  is 1, then we have 75 % permission on  and . Finally, the predicate  also states that  must contain a valid proof number.

In Java, the logical way of creating new witness objects is to have an overloaded constructor for every proof script. That is not possible in Chalice, so instead we generate a factory method with a unique name for every proof script. These methods are placed in the class that calls them. Thus, in our case, the factory method  , which requires the permissions and properties used in the proof and ensures a magic wand witness, is put into the  class.

We do not include a full listing of the generated Chalice code; however, it can be generated using the online version of the VerCors tool set [36].

### Correctness of the encoding

When Chalice is used as the back end, the semantics used for formulas is Implicit Dynamic Frames with fractional permissions. This logic does not define a magic wand and, therefore, the magic wand is no more than syntactic sugar for our encoding. The obvious question is if the magic wand as we implement it really is the same magic wand as in separation logic.

In this section, we provide an argument as to why our encoding is a correct implementation of the magic wand. We will do so based on the view that a static verifier is a tool that, given a program with specifications, establishes the existence of a correctness proof of those specifications. For example, for a sequential program it would establish the existence of a Hoare logic proof. Therefore, we will show that the program before the witness transformation can be proven correct if and only if the program after the witness transformation can be proven correct.

The result is that our encoding correctly implements the magic wand of any logic whose semantics extends that of IDF and in which the two proof rules for the magic wand that we use are sound.

To avoid unnecessary clutter, we will ignore scoping and visibility rules for variables, as it is well known how to fix these issues. Moreover, we will focus on the places where magic wands are introduced and eliminated. In proofs, magic wands can also occur in many other places, as they are carried along in proofs and specifications. However, as long as a magic wand is not introduced or eliminated, there is no difference between it and any other formula and thus requires no special treatment, while showing the correctness of the encoding.

The existence of proofs in a proof system is denoted with the symbol $\vdash$. That is,

$$\begin{aligned} F_1 , \ldots , F_n \vdash G \end{aligned}$$

denotes that there is a proof that $G$ logically follows from $F_1 \mathrel {\star }\cdots \mathrel {\star }F_n$. The introduction and elimination rules for magic wands on which our tool support is based are the following [32]:These rules are used in low level proofs to show that one claim logically follows from the previous one. These low level proof can be used as part of Hoare Logic proofs by embedding them. For example, if we can derive $F \vdash G$ then we can write 
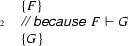
 Consider the following two fragments that use our **create** and **apply** annotations to reason about magic wands: 
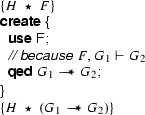
 and 
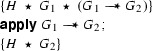
 The meaning of these fragments is, by definition, the same as the following two fragments that are motivated using the formal rules: 
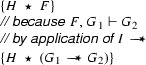
 and 
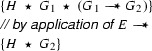


We show that we can eliminate the magic wand formulas from these fragments by transforming them into magic wand-free equivalents. We also show that each transformation step is reversible. As a first step, we lift the proof script and the application of the magic wand formula from the fragments into a separate class **Wand**:
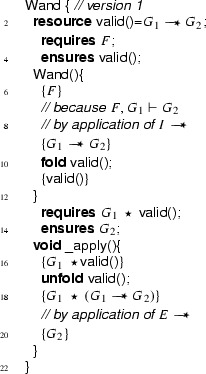


Using this class, the fragments can be rewritten to 
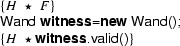
 and 
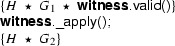
 Instead of using a magic wand in the fragments directly, we now have encapsulated the magic wand in the  predicate of the ghost class . Note how the resources needed to prove the magic wand ($F$) are explicitly required in the contract of the constructor. The reverse of this first program transformation step is inlining, which is known to be correct, so this first step preserves correctness of the annotated program.

The remaining occurrences of the magic wand are now in the ghost class , and in particular in the definition of the predicate . As a second step, we eliminate the magic wand from the definition of , by moving the proof of the magic wand from the constructor to the  method. The result is that instead of having a magic wand stored in the  predicate, we now have the resources needed to create the magic wand stored in the  predicate.
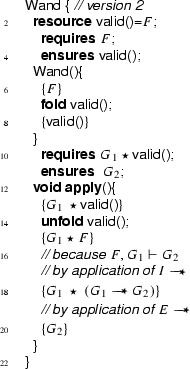


Clearly, since the specifications of the constructor and method  did not change compared to the encoding in version 1 of class , any client programs can be proven correct in the context of the first  encoding if and only if it can be proven correct in the context of the  version 2 encoding.

Finally, as a last step to get to the encoding that we have actually presented above, we can let the magic wand introduction and elimination steps cancel each other and simplify the annotated body of the  method as follows: 
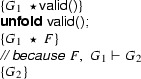
 This removes the last occurrence of the magic wand from the annotated code.

We have sketched how a program correctness proof of a specified and annotated program before the witness encoding can be transformed into a program correctness proof of the same program after the witness encoding and back. This means that if we start with a specified program, apply the witness encoding to it and then use Chalice to show that there exists a proof of the transformed program, we can transform the proof whose existence was proven by Chalice back to a proof of correctness of the original program. Hence, our encoding is correct.

The transformation is sound and complete with respect to the specifications and annotations on the original program. This does not mean that our transformation is complete for all programs that have been specified with magic wands. There might be a specified program, which is correct, but which cannot be proven correct using our method. To prove the absence of such programs, i.e., completeness of the annotations and encoding, several proof theoretic questions would have to be answered. For example, the proof rules for magic wand would have to be proven complete and also a cut elimination theorem would have to be proven.

For this correctness sketch, we have conveniently forgotten about visibility of fields and methods. However, the actual implementation does take care of those. Similarly, the implementation takes care of multiple proofs by having multiple factory methods instead of a single constructor and using a case distinction in the bodies of the  predicate and  method to distinguish between the different proof resources and proofs.

### Recipe for the encoding

Next, we sketch the complete translation algorithm from a program that has been annotated using magic wands into a program that is annotated using witnesses. This requires to translate all occurrences of magic wand formulas and all occurrences of magic wand proof scripts. In this sketch, we use the notation $\overrightarrow{\alpha }$ to abbreviate the list/vector $\alpha _1 , \ldots , \alpha _n$.

The implementation supports magic wand formulas where both the required formula and the ensured formula are a separating conjunction of predicate invocations. For this presentation, we limit ourselves to a single required predicate and a single ensured predicate. We can do this without loss of generality because any formula can be turned into a single predicate formula, where the predicate body is the separating conjunction of the individual formulas and where the parameters are the free variables in the formulas.

**Fig. 16:**
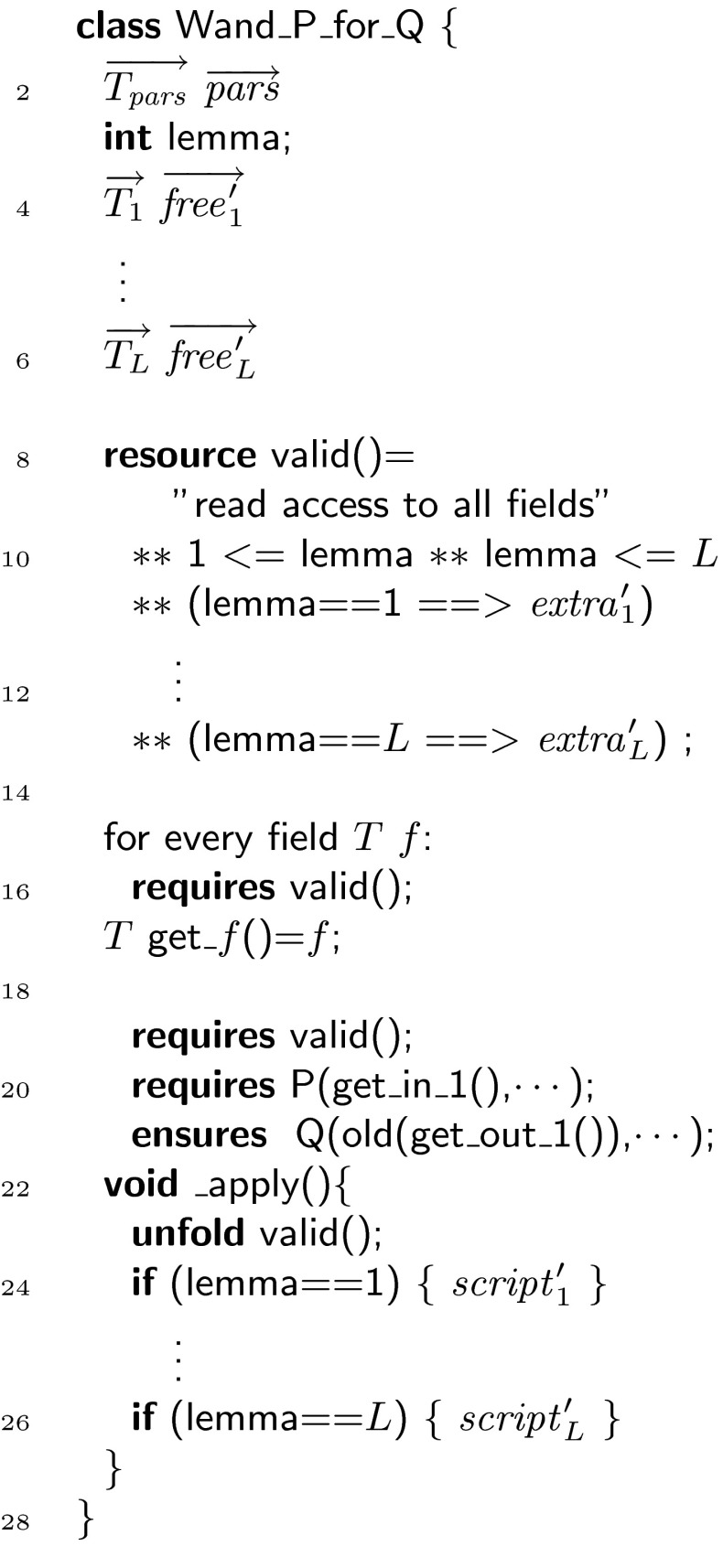
Template for a witness class

Thus, we assume magic wands of the form: 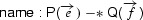


For every magic wand formula that uses the same combination of predicates, we use the same witness class, whose template is given in Fig. 16. First, we generate a list of field declarations for the witness class, declaring all the parameters used in the magic wand formula:

- for $$i = 1 \cdots |\overrightarrow{e}|$$: $$ typeof (e_i)$$  $$i$$;
- for $$i = 1 \cdots |\overrightarrow{f}|$$: $$ typeof (f_i)$$  $$i$$;

where $typeof$ is a meta-operator that extracts the type of an expression.

Furthermore, the witness class defines getters for all of its fields. This allows us to replace the magic wand formula 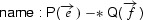 with a formula that states that  is a valid witness of this formula: 
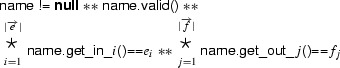


Note that, the quantifiers in this formula have to be expanded at code generation time because they use mathematical meta-notation that is not part of our syntax.

The annotated program will contain several proof scripts to create a witness, all matching the following template. 
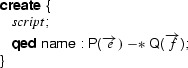


Let the total number of proof scripts be $L$, and let the scripts be numbered $1,\ldots ,L$.

**Fig. 17:**
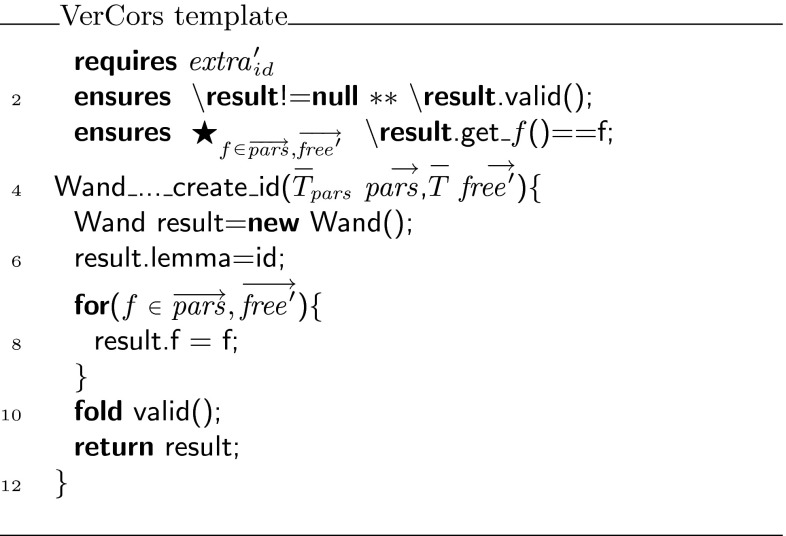
Factory method for a witness

For each script, we compute the list of free variables used in the script and/or the wand formula ($\overrightarrow{ free }$) and their respective types ($\overrightarrow{T}$). The list of free variables by definition contains **this**. To avoid name clashes, we prime all variable names, resulting in the list $\overrightarrow{ free '}$. Each proof script is then replaced with a call to a factory method, using an appropriate proof script identifier  to generate a unique name, as defined in Fig. 17: 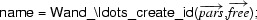 The given proof script $$ script _{id}$$ is used to construct the formula $$ extra '_{id}$$ and the proof script $$ script '_{id}$$, as follows:

- For every occurrence of **use** $$\phi $$ in $$ script _{id}$$, a conjunct $$\phi '$$ is added to $$ extra '_{id}$$.
- Every other proof hint is renamed by priming all variables and then added to the proof script $$ script '_{id}$$.

When turning magic wands into witnesses, witness variables must be declared for each usage of the magic wand formula.

- when used in **requires**, the variable is declared in the **given** of this method;
- when used in **ensures**, the variable is declared in the **yields** clause of this method; and
- when used in **witness** or **loop_invariant**, the variable is declared as a local variable.

Finally, every application of a magic wand 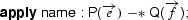 is replaced by a call to the  method of the witness: 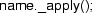


This completes the description of the encoding, as it is implemented.

### Applicability of the transformation

We have described two transformations: one that introduces witnesses for predicates and one that introduces witnesses for magic wands. Both transformations are implemented as separate passes in the VerCors tool. The tool first applies the magic wand transformation and then it applies the predicate transformation. As a result of this, the tool can deal with predicates that do not use magic wands in their definitions and magic wands defined on top of those predicates. To verify larger systems, this might not be enough. One could imagine that on top of the magic wands, there would be another layer of predicates, and maybe on top of that even another layer of magic wands. A typical example of this situation would be when we use a predicate to hide the fact that a class wraps another class, and that other class uses magic wands in its contracts. We can easily extend the tool to support this alternation of predicates and magic wands by alternating the encodings and in each step encoding the outermost layer of predicates or magic wands. However, note that this process is not able to deal with an inductively defined predicate, where the predicate occurs nested inside a magic wand, such as in the following (contrived) example: 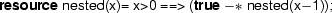 It is an open question if the two transformations can be merged in order to be able to deal with this pattern.

Our implementation of the transformations was tested in combination with the Chalice tool. In principle, both transformations can also be used in combination with VeriFast as a back end. In practice, it is not a good idea to use the witness transformation for predicates for VeriFast because VeriFast can deal with those predicates itself and thus more effectively. However, in the case of alternating predicates and magic wands, there has to be a way for the outer predicate to manage the inner magic wand witness, which requires the use of witnesses for those predicates. It is ongoing work to implement VeriFast as a back end and to search for a better solution.

## Magic wand examples

This section presents two more involved examples that use the magic wand in their annotations. First, we consider the tree delete challenge. Second, we consider an iterator protocol for iterators on a list of integers. For both examples, we show how to provide full annotations, so that they can be verified by the VerCors tool set.

### Verification of the tree delete challenge

**Fig. 18:**
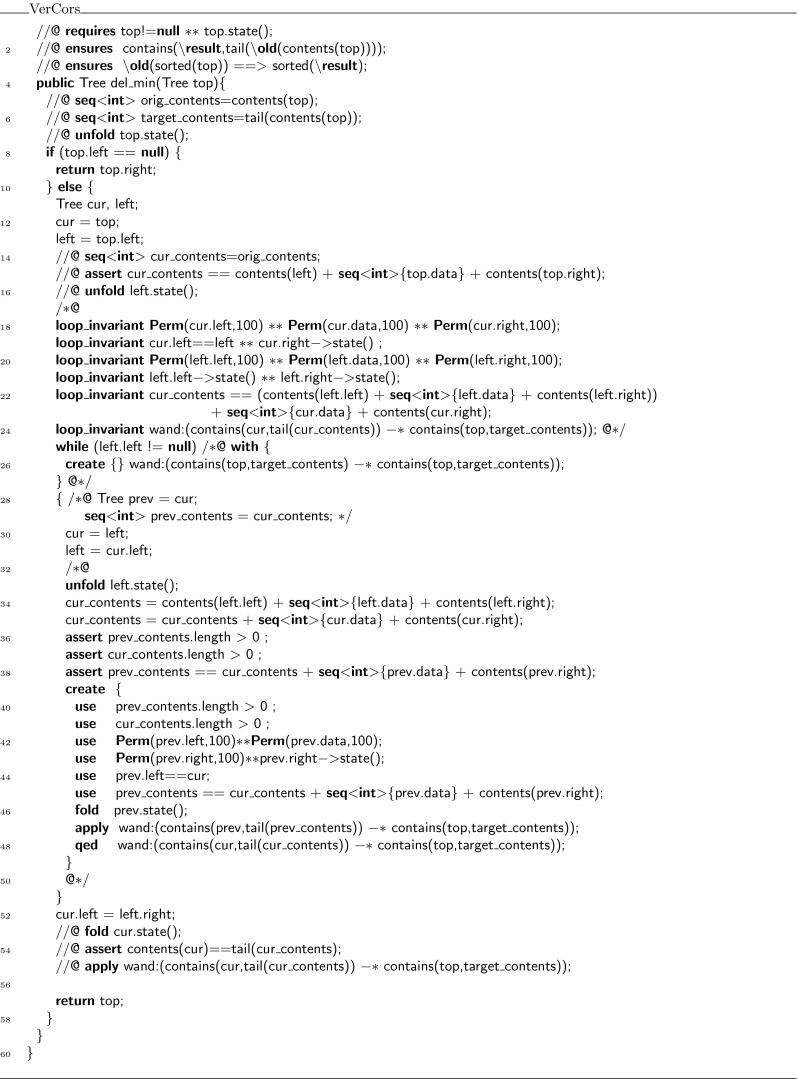
Fully annotated version of iterative tree delete algorithm

Using the encoding, we can verify the tree delete challenge. Taking the annotated algorithm in Fig. 4 as a starting point, we have to provide proof scripts whenever we create a magic wand formula to make it verifiable. The resulting fully annotated version of the tree delete algorithm can be found in Fig. 18.

Since the magic wand formula is used in the loop invariant (and a new instance of it is needed with every iteration of the loop), we actually require witnesses for the creation of magic wand objects in two places in the annotated program: we need a witness to create a magic wand formula to show that the loop invariant holds upon loop initialisation, i.e., before the loop is actually executed (see line 26), and in addition we need to provide a witness to create a magic wand formula inside the loop body, to show that every iteration of the loop preserves the loop invariant (see lines 39–49). In each of the places where the apply method is used (see Lines 47 and 55), it could be either of the proofs that is used (depending on how many iterations, the loop needs). Hence, it would not be possible to encode each proof in a separate class.

The online version of the VerCors tool set [36] can be used to inspect the full Chalice encoding of this example. Using the Chalice encoding, the VerCors tool can verify the iterative tree delete algorithm without any problems. The entire example verifies in 13 min on an Intel i7-2600 (3.40 GHz).

### The iterator protocol

As a second example, we present a variant of the iterator protocol from Haack and Hurlin [9]. To simplify our presentation, we have chosen to work with a list of integers rather than a list of objects.

**Fig. 19:**
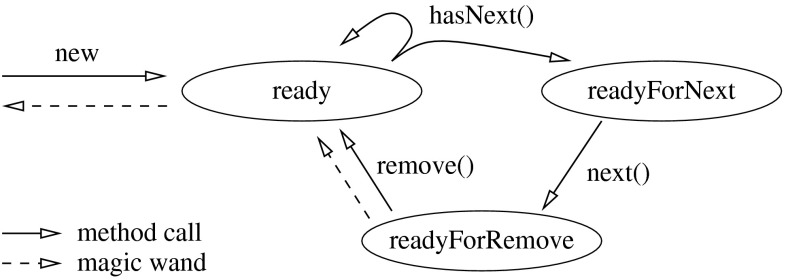
State machine for the iterator protocol

The iterator protocol uses the following three states: ,  and . The entire protocol is displayed in Fig. 19. When an iterator is created, permissions on the current list are folded in the  state. In this state, one may apply a magic wand to recover the permissions on the current list, or one may call  to test for the existence of a next element. If such an element exists, the next state is , otherwise the next state is . If the state is , the  method can be used to retrieve the current element and the next state will be . In the  state, one can use either the  method or the magic wand provided by  to go back to the  state.

**Fig. 20:**
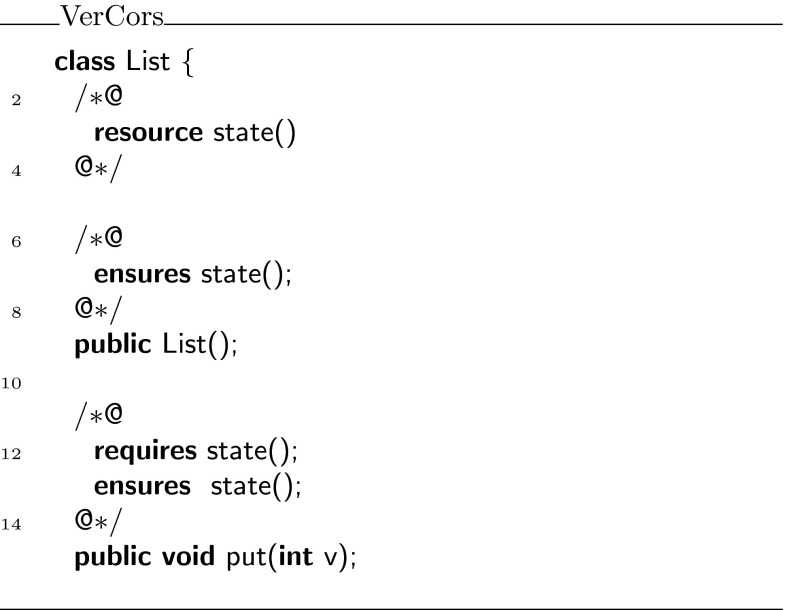
Specification of an integer list

**Fig. 21:**
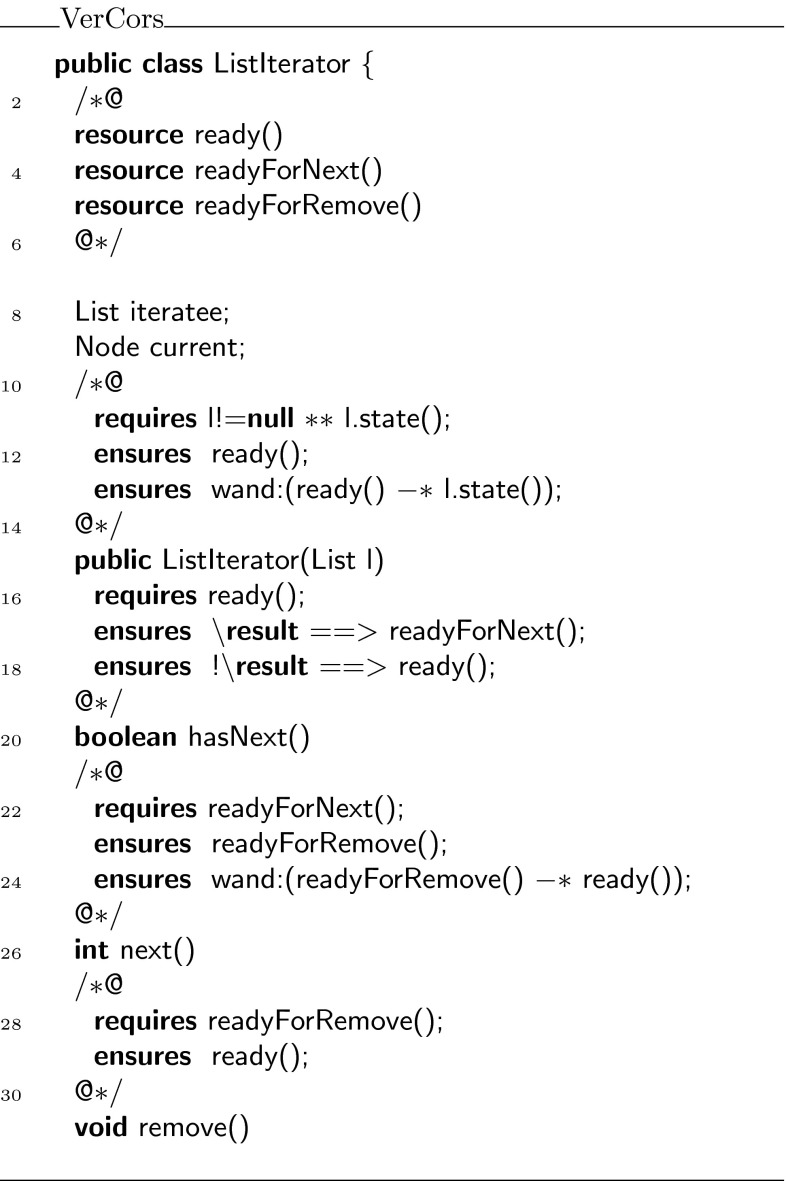
Specification of the list iterator

The specifications of the integer list and the list iterator can be found in Figs. 20 and 21, respectively. We have annotated implementations of these interfaces, which have been verified with the VerCors tool set. The fully annotated versions are available from the same website as the tree delete example.

**Fig. 22:**
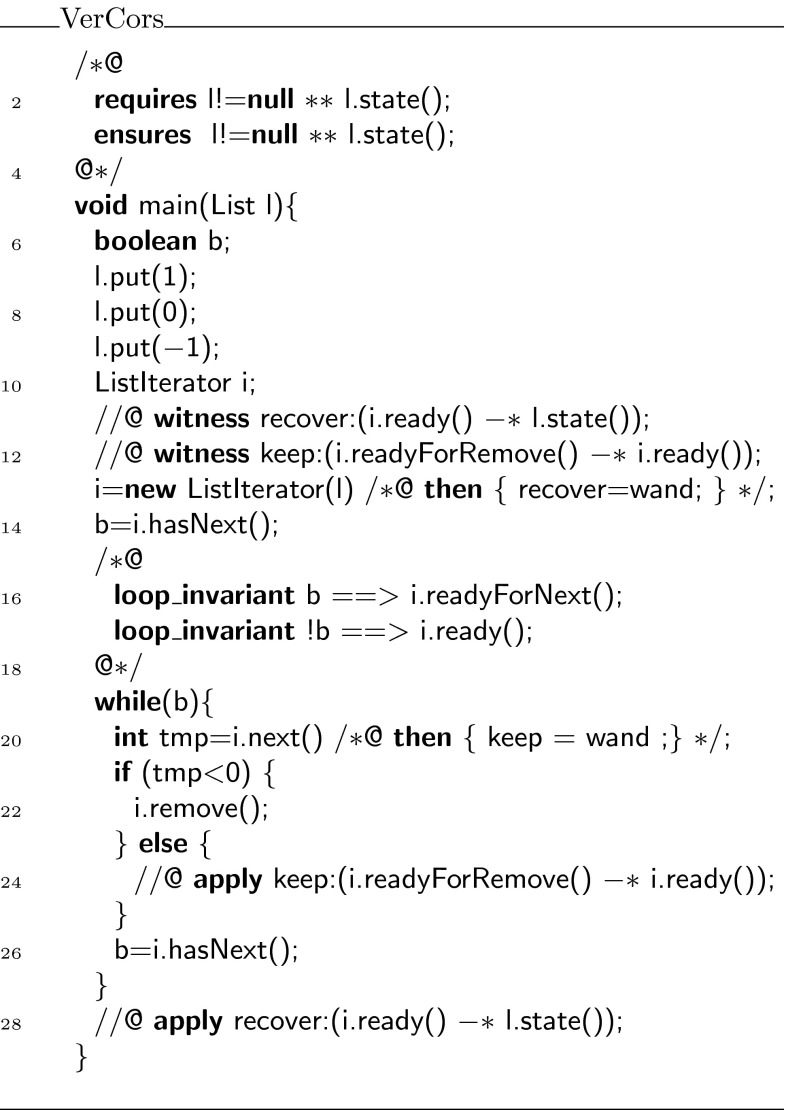
Example that uses the list and iterator

We have also verified a small example that illustrates the usage of the list and the iterator, see Fig. 22. In this example, we create a list containing $[-1,0,1]$ and then use an iterator to remove the negative elements. The entire example verifies in 19 s on an Intel i7-2600 (3.40 GHz).

## Conclusions

In this paper, we have introduced two witness transformations from Java with separation logic annotations into a form that can be checked with Chalice. The first transformation replaces predicates with parameters by witness objects with parameter-free predicates (except for the implicit **this** parameter). The second transformation replaces magic wands by witness objects. To overcome undecidability when reasoning about magic wands, the user has to provide a proof script that can be used to check that resources are indeed correctly exchanged by the magic wand. Both transformations are not Chalice-specific, i.e., in principle they can be used as an encoding for any object-oriented language with separation logic annotations. This is reflected in the implementation, which will remove the witness extensions and replace them with objects and simpler predicates. In this process, unknown expressions and annotation are simply copied, which means that the current implementation will work for other similar back ends without modification. However, it might require additional or different proof hints.

In this paper, we have shown that the transformations that we define are sound and complete, i.e., the original specification is correct, if and only if the transformed specification is correct.

It should be emphasized that all examples in this paper have been machine checked, however, we feel it is too early to make any claim about the class of programs that can be validated with our approach: the fact that a proof exists does not always mean that it can be found automatically by a prover.

To illustrate our approach, we have presented two larger examples: the tree delete example, which demonstrates how magic wands can be used in loop invariants, and the iterator example, which shows how a magic wand is used to enforce that method calls happen in the prescribed order. Both examples with full annotations are automatically verified by the VerCors tool set. In this paper, we have not presented an example that uses both witness transformations at the same time, but such an example is available online [36].

### Related work

*The tree delete challenge* Our use of the magic wand to verify the iterative implementation of the tree delete algorithm is just one of the ways of solving the tree delete challenge. Another way of solving the problem is using Türk’s rule for loops [37], which effectively offers the possibility of writing loop annotations as if the loop were a recursive function. In other words, the loop body can contain a block of proof hints, whose application is delayed, forming a stack of delayed proof steps. When the loop exits, all delayed steps are applied. As these steps can implicitly set aside resources too, each delayed step is equivalent to a magic wand. The advantage of using magic wand syntax rather than the Türk rule is that magic wands can be used in any location in the code, whereas the Türk rule is only applicable to loops. Yet another mechanism for specifying the iterative version of the tree delete problem is using *specified blocks* [38]. The Krakatoa tool [39] implements this generalization of the Türk rule, which allows attaching pre- and postconditions to arbitrary statement blocks, instead of just to loop bodies.

The tree delete challenge can also be addressed using the Zipper data structure from functional programming [40]. This is an alternative way to treat the shift of focus: with little effort it allows to write the invariant in such a way that in each iteration the focus is shifted by one step. Once the left-most node is removed, the Zipper structure allows to move the focus back to the root. To use a Zipper structure, one would have to write the basic data structure, which requires quite a few lines of specification, but which can easily be automated. For those cases where the pre-defined functions work, the annotation work load would then be limited to a few instructions that move the focus. However, if a non-standard move is required, it would have to be spelled out completely at a considerable specification effort, because only pre-defined moves would be automatically generated.

*Tool support* While jStar does not implement the magic wand at the moment, the coreStar [41] framework, that is used to implement it, is in principle capable of supporting the magic wand and its proof rules.

The VeriFast tool implements lemmas [13], which are equivalent to a subset of magic wands. That is, lemmas can transform sets of resources, just like magic wand can. But magic wands can exchange resources as well, for example, a magic wand can express the capability to exchange a read-only permission for accessing a location on the heap to a full write permission for the same location. If one were to express this using a lemma, the lemma would have to be paired with a predicate that captures the extra permissions to match the functionality of the magic wand.

Jost and Summers [25] have written a tool that translates VeriFast specifications to Chalice. They analyze VeriFast predicates to see if they can be split into a parameterless permission predicate and a boolean function. If such a split is impossible they resort to use ghost variables in the object to represent the predicate parameters. The advantage of such an approach is that it leads to a simpler encoding that requires less annotations to be verified. The disadvantage is that the encoding does not work if the same predicate must be held more than once on the same object, whereas our encoding supports this. Their approach could be extended to support multiple predicates using multiple sets of ghost variables, but then one would have to keep track of which set of variables is used making their encoding more complicated than ours.

Schwerhoff and Summers [22] more or less simultaneously have implemented support for magic wands in the Silicon verifier. Their support is very similar in the sense that we both use the idea to apply a magic wand (keyword **apply**). But in their approach, creating a magic wand (keyword **package**) is based on automatically computing the extra resources of a magic wand, whereas our **create** blocks require the user to explicitly specify those (using the **use** keyword). However, the other proof script statements in our **create** blocks could be translated to **package** expressions automatically. This means that a Silicon back end for the VerCors tool could offer the best of both worlds: simple magic wand instances can be handed off to Silicon directly, and in cases that Silicon cannot deal with a magic wand automatically then our more detailed witness annotations and transformations can be used to guide the Silicon prover through the difficult proof. We are currently investigating this idea in more detail.

Automated proving for special cases of the magic wand is being worked on. For example, in the setting of the logic of boolean bunched implications (a close relative of separation logic), Park et al.  [42] have given an algorithm for deciding the validity of a formula with magic wands. Furthermore, Atkey has shown that for a restricted magic wand syntax it is possible to directly derive verification conditions [8]. Formally, the fragment used in that paper is incomparable to ours: magic wands may not occur nested on the left-hand side of another, which is allowed but not implemented for our encoding, but it is allowed to use disjunction in certain places, which our fragment does not allow. Moreover, the paper focusses on statements about execution time and memory usage and not functional verification in general.

The transformation that turns magic wands into objects is a variant of defunctionalization of closures in a functional programming language (see for example [43]). Defunctionalization transforms higher order functions (functions that take functions as arguments) into normal functions. A magic wand is a function that operates on permissions (that may be executed at most once). This makes the **apply** operator a higher order function and subsequently our encoding of the apply function ends up being an instance of defunctionalization.

Finally, the specification style of the generated object, defining a  predicate is inspired by the standard methodology for Boogie of Barnett et al. [44].

### Future work

*Annotation generation* Clearly, the major drawback of our approach is the large number of (long) annotations that the user has to provide at the moment. To address this issue, we will first of all investigate heuristics to come up with good default specifications. We will also investigate if methods for automatically deriving specifications can be adapted to our situation. For example, it might be possible to use the constraint-solving algorithm developed by Ferrara and Müller [45] to infer a large number of the witness management annotations.

In addition, as mentioned above, the approach taken by Jost and Summers in their translation of VeriFast to Chalice can deal with a number of cases with less annotation load. We will study if we can integrate their work, so that the simple cases can take advantage of their encoding, while the encoding of this paper can be used for more complicated cases.

The current implementation of the witness transformation for magic wands requires that all the resources that are stored inside the magic wand are explicitly mentioned using the **use** keyword. The problem of finding out which resources have to be stored is similar to the problem of finding out which resources to pass to a method during a call and which resources to keep. Therefore, we will study the techniques for solving this, such as the use of frame inference and bi-abduction [46], and see if they can be reused and/or adapted to our approach.

*Extensions* The witness encodings presented in this paper require verification tools that support the **Perm** predicate of separation logic. These basic permissions can be encoded by a verifier as a map from locations to fractions, representing the fractions held. We believe that by adding such a map to our encoding, we create a transformation from programs specified in separation logic to programs specified in first-order logic. This would make it possible to reuse existing first-order tools. In the case of static checkers, this might not be very practical due to the added annotation workload. However, by exploiting existing run-time checkers for JML and reflection in Java it should be possible to put together a run-time checker for separation logic specifications. In this case, no extra annotations are needed because formulas can simply be evaluated.

The witness transformations proposed in this paper transform the formulas and proof script annotations to get rid of predicate arguments and magic wands completely. This allows us to use back ends that do not support those features at all. However, there are also verifiers that can be used as a back end that support some features at least partially. By putting in intermediate stages in the transformation, we may be able to exploit those features. E.g., VeriFast supports predicates with argument and lemmas. Thus, we could add an intermediate step in which each magic wand is translated into a pair of a predicate and a lemma. This would allow us to pass a version to VeriFast with less encoding, which might be more efficient than a fully encoded version.

Finally, we are currently investigating how permission-based separation logic can be used to reason about Scala programs. This requires the possibility to specify the behavior of closures. We are investigating, if we can extend our approach also to encode closure specifications.

## Footnotes

1 We also considered VeriFast as a back end, but this would require more specification rewriting due to the strict separation between program state and logical state in VeriFast.

2 So named because we disallow disjunctions and because we suspect that all formulas in this logic are *supported* [27].

3 We chose this protocol for illustrative purposes, not because of its practical value. However, more meaningful protocols can be treated in a similar way, as shown in Sect. 6.2.
